# Automated Long-Term Monitoring of Parallel Microfluidic Operations Applying a Machine Vision-Assisted Positioning Method

**DOI:** 10.1155/2014/608184

**Published:** 2014-07-15

**Authors:** Hon Ming Yip, John C. S. Li, Kai Xie, Xin Cui, Agrim Prasad, Qiannan Gao, Chi Chiu Leung, Raymond H. W. Lam

**Affiliations:** Department of Mechanical and Biomedical Engineering, City University of Hong Kong, Kowloon, Hong Kong

## Abstract

As microfluidics has been applied extensively in many cell and biochemical applications, monitoring the related processes is an important requirement. In this work, we design and fabricate a high-throughput microfluidic device which contains 32 microchambers to perform automated parallel microfluidic operations and monitoring on an automated stage of a microscope. Images are captured at multiple spots on the device during the operations for monitoring samples in microchambers in parallel; yet the device positions may vary at different time points throughout operations as the device moves back and forth on a motorized microscopic stage. Here, we report an image-based positioning strategy to realign the chamber position before every recording of microscopic image. We fabricate alignment marks at defined locations next to the chambers in the microfluidic device as reference positions. We also develop image processing algorithms to recognize the chamber positions in real-time, followed by realigning the chambers to their preset positions in the captured images. We perform experiments to validate and characterize the device functionality and the automated realignment operation. Together, this microfluidic realignment strategy can be a platform technology to achieve precise positioning of multiple chambers for general microfluidic applications requiring long-term parallel monitoring of cell and biochemical activities.

## 1. Introduction

Due to the technology advancements in microfluidics over the past two decades, the applications have extended to cover cell culture [1–6], cell analysis [7–10], drug testing [11–13], DNA analysis [14–18], crystallization [19, 20], and liquid chromatography [21]. In particular, microfluidics offers outstanding capabilities for the high-throughput mammalian cell culture, mainly because microfluidics can essentially define their required growth conditions for cell culture, separation, and identification with higher accuracy and sensitivity [22]. The parallel cell applications can be further integrated with programmable components [23–26] and biosensors to perform real-time sensing abilities (e.g., dissolved gases and cell density measurements) [27–29]. Pneumatic valves [30], pumps [30], and mixers [31, 32] are examples of the basic microcomponents which can automate the fluid-handling operations, for example, flow direction and flow rate as well as solution concentration. Gómez-Sjöberg et al. developed a fully automated cell culture system on an integrated microfluidic chip which contains 96 chambers with time-lapse monitoring [33]. Skafte-Pedersen et al. built a programmable microfluidic platform with real-time optical read-outs to perform parallel cell culture experiments [34].

Monitoring of parallel microfluidic operations can be achieved by connecting the microfluidic device to a high-end automated positioning system. This long-term process requires stable physical conditions such that samples in the microchambers are at the expected position (within precision of a few microns or smaller) and in focus for the imaging or other recording schemes. Misalignment of the detection regions may lead to failure of the microfluidic devices that their recorded information is incomplete and insufficient. For instance, the displacements can be attributed to position errors of the moving components, external forces from the peripherals such as tubing for fluid connections, and deformation of device materials (e.g., polydimethylsiloxane (PDMS) elastomer). In view of the accumulation of possible device displacements during device movements and microfluidic operations, precise positioning of the device regions is an essential requirement to achieve automation of high-throughput microfluidics for the long-term monitoring of parallel microfluidic operations.

In this paper, we introduce the application of micropatterned alignment marks located in a microfluidic device to achieve position control of microfluidics and parallel monitoring of activities in multiple microchambers. The microfluidic device is fabricated by multilayer soft lithography [30, 35] for PDMS, which is a widely used material for microfluidics largely because of its biocompatibility and inert chemical properties [36]. The alignment marks are located at defined device positions next to the monitored regions; thereby they are the reliable reference positions for the monitoring operations. We have developed an automated position alignment system for the long-term monitoring of parallel microfluidic operations. We have also designed and fabricated a multichamber microfluidic device which contains 32 (4 rows × 8 columns) chambers. An alignment mark was fabricated at a defined position next to each chamber. The alignment mark can be recognized for its position and orientation by real-time microscopic imaging and image processing. Position control of the microchambers can then be implemented by movements of the motorized stage. We performed experiments and analyses to examine the performance of the automated positioning operation using the microstructures in microfluidics as alignment marks. Additionally, we performed further experiments to demonstrate the applicability of the microfluidic parallel monitoring technique in high-throughput analyses of cell growth behaviors.

## 2. Methods

### 2.1. Automated Platform for Microfluidic Operations

**Figure 1:**
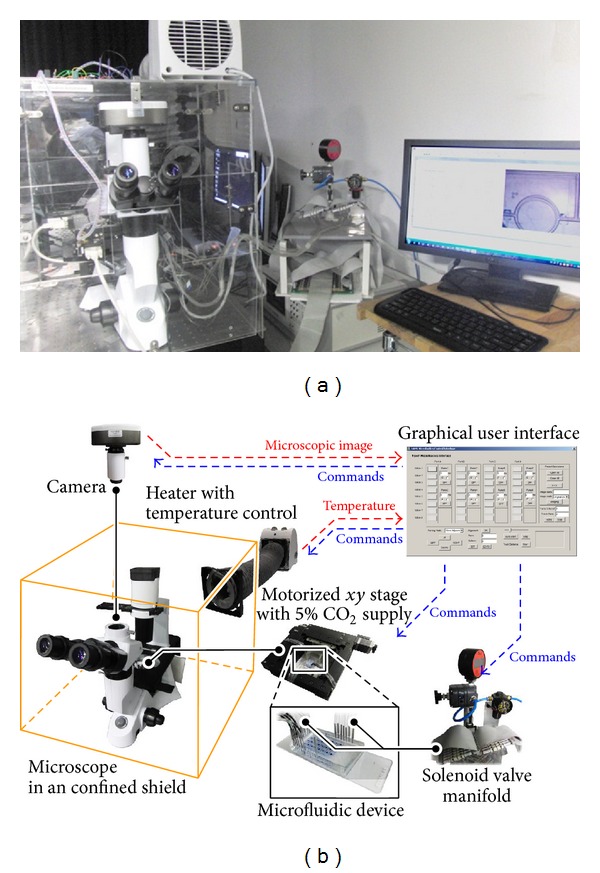
(a) Photograph and (b) schematics of the automated microfluidic parallel monitoring platform.

We established an automated platform to control microfluidic operations (Figure 1(a)). This platform included an inverted microscope (PW-20 BDS500EPI, Proway Optics and Electronics, Ningbo, China) placed in a confining shield with the capabilities of temperature, humidity, and surrounding gas (e.g., 5% CO_2_ in air for cell culture applications) controls (Figure 1(b)). The temperature control was achieved by first measuring temperature in the confined shield with a temperature sensor (LM35, Texas Instruments, Dallas, Texas, United States). The sensor signals were then passed to a temperature controller consisting of a microprocessor (Atmega16, Atmel, San Jose, California, USA) for computing the driving voltage for a heater (NT20-12D, Midea, Hong Kong) based on a proportional-derivative feedback control scheme, in order to maintain temperature in the confined shield. The stabilization and homogeneity of temperatures at different positions of the microfluidic device were validated by an experiment described in Figure S1 in Supplementary Material available online at http://dx.doi.org/10.1155/2014/608184. The gas condition and humidity were conditioned by continuously flowing the desired gas (compressed air mixed with 5% CO_2_) through a water tank located next to the microscope for humidification (Figure S2a). The humidified gas then passed into the incubator region surrounded by another inner confining unit moving with a motorized *xy* stage (Figure S2b) installed in the microscope. The motorized stage contained a customized device holder for the controlled movement of microfluidic devices. A cooled CCD camera (TCH-5.0ICE, Xintu Photonics, Fuzhou, China) connected to the microscope for monitoring and recording experiment processes. As this automated platform mainly focused on the microfluidic devices driven by the pneumatic valves based on multilayer soft lithography [30], it included up to 96 solenoid valves to achieve programmable air pressures from a common compressed air source. These 96 solenoid valves were driven by a computer, in which the commands were transmitted via an interface card (PCIe-DIO96H, Measurement Computing, Norton, MA) installed. In this case, we could define the gas lines pressurizing valves in a microfluidic device, through Tygon tubing (inner diameter: 0.5 mm; Cole-Parmer, Vernon 24 Hills, IL) and stainless steel adaptors (outer diameter: 0.7 mm; New England Small Tube, Litchfield, NH) to inlets of the device. It should be noted that the outer diameter of the adaptors should be chosen to be bigger than inner diameters of the tubing and holes on the device (0.5 mm) to ensure perfect sealing against leakage. The tubing sections close to the microfluidic device were mounted on and moving with the motorized stage during operations; therefore device misalignment due to pulling of tubing during stage movement was negligible. Furthermore, we developed a customized graphical user interface to monitor the platform status (e.g., microscopic view and temperature) and control all of the* xy* stage, camera, heater,and solenoid valve operations.

### 2.2. Fabrication

A microfluidic device was fabricated based on the multilayer soft lithography [30]. First, three molds for (1) the control layer, (2) the flow layer, and (3) the microwell layer were fabricated by photolithography for photoresist microstructures patterned on silicon wafers. The control layer mold contained a 10 *μ*m thick layer of SU-8 negative photoresist (Microchem, Newton, MA) on a silicon wafer. The flow layer mold contained a 10 *μ*m thick AZ4620 positive photoresist (AZ Electronic Materials, Branchburg, NJ) layer on a silicon wafer, followed by the reflow process for rounded channel cross-sections. Further, the microwell layer mold consisted of two layers of SU-8 microstructures on a silicon wafer. The upper 100 *μ*m thick SU-8 layer was the chamber structures while the lower 10 *μ*m thick SU-8 layer contained the alignment mark patterns. After the mold fabrication, all of the three molds were silanized with a high-molecular weight trichloro-perfluorooctyl saline (Sigma-Aldrich) to facilitate the polydimethylsiloxane (PDMS: Sylgard 184, Dow Corning, Midland, Michigan) release. Then, we fabricated a three-layer microfluidic device for parallel microfluidic operations based on multilayer soft lithography [30]; and the more detailed fabrication process is shown in Figure S3.

### 2.3. Liquid Replacement in Microchamber

We injected a dye solution and water into a microchamber alternatively with different flow rates and captured videos for the liquid replacement processes in the chamber using an inverted optical microscope. We then extracted image frames from the videos. The images were processed by a customized script written in MATLAB (version R2010b, Mathworks, Natick, MA) to quantify the results and obtain the relationship between the liquid content, flow rate, and injection time.

### 2.4. Hardware for Image Processing

We have utilized a graphic processing unit built in a graphic acceleration card NVIDIA (GeForce GT 640, NVIDIA, Santa Clara, CA) to speed up the image processing in our microfluidic position control system. Specifically, we applied CUDA, a parallel computing architecture in order to greatly reduce the processing time. Such improvement is mainly based on the optimization of (1) memory usage, (2) parallel execution, and (3) instruction usage. The balancing of these three terms directly determines the computation efficiency. Instead of implementing an optimized image processing algorithm, which focuses only on reducing the number of processed pixels by more complicated calculations, CUDA considers both the cost of algorithms and the number of processing units. For instance, using CUDA, the overall image processing time for the automated chamber positioning in this work reduced from 20389 ms (without CUDA) to only 452 ms.

### 2.5. Cell Culture and Seeding

BALB/3T3 murine embryonic fibroblasts (CCL-163, Advanced Type Culture Collection (ATCC)) were cultivated in Dulbecco's Modified Eagle Media (Gibco, Lift Technologies, New York, USA) containing 10% fetal bovine serum. The cells were cultured in a 5% CO_2_ humidified incubator at 37°C. When the cells grew until confluent, they were trypsinized with 0.25% trypsin in ethylenediaminetetraacetic acid (EDTA, Sigma-Aldrich) and passaged at a cell density of 3000 cells/cm^2^, manipulated under a sterile tissue culture hood.

The microfluidic devices were sterilized by flushing the flow channels with 70% ethanol and 1x phosphate-buffered saline (PBS). The flow channels were subsequently precoated with 20 mg/mL human fibronectin (Sigma-Aldrich) in 1x PBS for 1 h to promote cell adhesion and growth, followed by replacing the liquid with pure 1x PBS and then the culture media. Afterward, the 3T3 cells at a density of ~10^5^ cells/mL were loaded into the microchambers sequentially. The procedures for liquid replacement and cell seeding are described in Section 3.3.

## 3. Results and Discussion

### 3.1. Device Configuration

**Figure 2:**
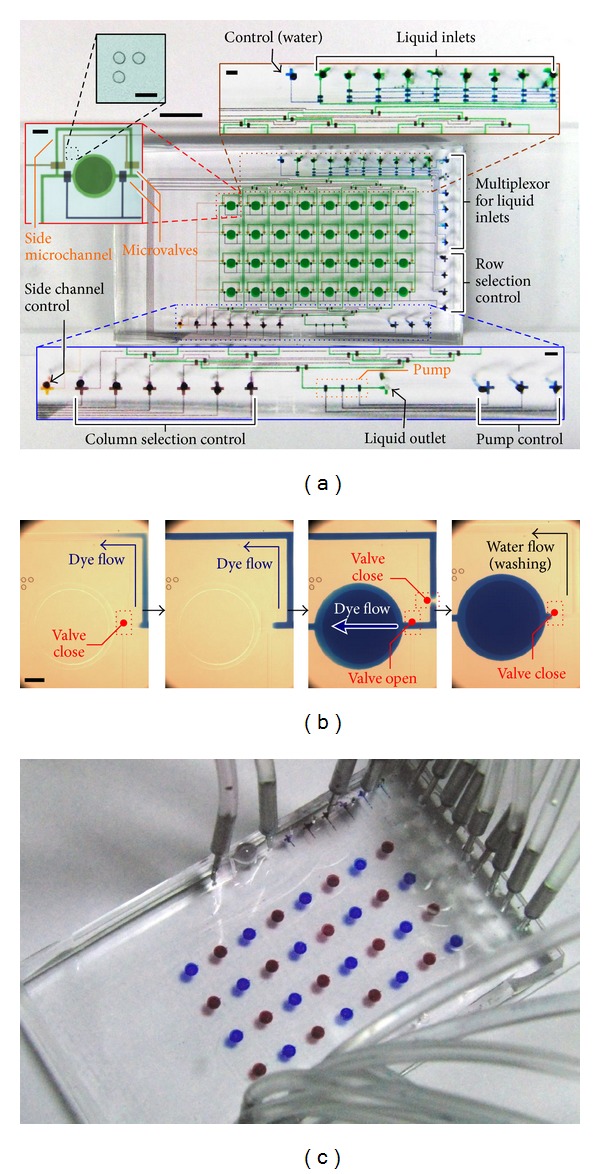
(a) Device configuration of a 32-chamber device. The channels have been filled with dyes for better visualization.* Left* inset (scale bar: 500 *μ*m): microchamber region including an alignment mark (*upper left* inset; scale bar: 250 *μ*m), a side channel with its control valves, and the row-selection valves. The darker circle in the chamber indicates a deeper microwell.* Upper* inset (scale bar: 500 *μ*m): liquid multiplexor for selection of one inlet liquid from a total of eight choices and upper multiplexor for column selection.* Lower* inset (scale bar: 500 *μ*m): lower multiplexor for column selection, side channel control, peristaltic micropump, and liquid outlet. Scale bar in the whole-chip photograph represents a length of 5 mm. (b) Procedures of dye injection into a chamber and the following washing step. Scale bar: 250 *μ*m. (c) A checker pattern of dyes with two different colors injected in the microfluidic device.

We developed a microfluidics device (Figure 2(a)) to examine performance of the position control strategy. The main objective of this device was to deliver liquids into one of the 8 × 4 microfluidic chambers in configured sequences, in order to achieve the defined cell or chemical analysis applications. This device consisted of a control channel layer and a flow channel layer containing microwells to induce heights of the chamber regions, manufactured from top to bottom.

The flow channel layer contained microchannels for fluidic connections and an array for 8 × 4 microchambers for high-throughput parallel operations. This device required 21 gas inlets with a common pressure of ~15 psi for all of the required valve operations. Microwell structures with a common depth of 100 *μ*m were located at each chamber. A side microchannel was designed next to the culture chamber (*left* inset in Figure 2(a)); thereby water and sterilizing agents could flow around the chambers before and after each medium/reagent insertion to the chambers. This operation was an important process to minimize any possible contamination in the chambers. A microfluidic pump was located at the downstream outlet channel of the device for liquid delivery. Selection of the inlet liquid was implemented by multiple microvalves. As shown in the* upper* inset in Figure 2(a), a microfluidic multiplexor and a separated microvalve (the* leftmost* channel) were used to define the opening of the nine inlet channels. In addition, sets of microvalves were applied to identify the channel openings for the target chamber for liquid insertion. We designed another multiplexor to control fluidic access of microchannels connecting to a specified column of chambers, while four rows of microvalves were configured to identify accessibility of a chamber row. Control lines with a common gas pressure (*leftmost* port in the* lower* inset) determined the openings of all side channels around the chambers. It should be noted that either the side channel valves or the row selection valves were opened during operation, because channel cleaning (side channels were opened) and medium/reagent insertion (chambers were opened) were two separated operations of this microfluidic device.

In addition, an alignment micropattern was fabricated right next to each culture chamber for position identification of the device during operations. The alignment mark consisted of three equal-dimension circles arranged as a right angle triangle (*upper left* inset in Figure 2(a)) such that the position and rotation of the device could be identified by an image-based position recognition algorithm. Overall, the microfluidic device was capable of liquid manipulation from a selected medium/reagent to every chamber and provided appropriate microenvironments in each chamber.

### 3.2. Microchamber Accessibility

An experiment was conducted to verify for the accessibility of injection of a selected inlet liquid into every microchamber in the device.* Blue* dye,* red* dye, and water (transparent) were used for visualization of liquid types from the inlets. As mentioned, the inlet liquid was specified by the inlet multiplexor and the opening of the chambers was defined by the row selection valves and the column multiplexor. The selected dye was injected into each column of chambers from* left* to* right* and into each chamber from* top* to* bottom* for the column. For each chamber, water and then the dye were firstly injected via the side channels around the selected chamber to ensure the selected inlet solution located at the chamber entrance (Figure 2(b), the two* left *subfigures). Following the opening switched from the side channel to the chamber, the dye then flowed into the chamber and the color change was observed (Figure 2(b), second* right*). Then, the opening switched back to the side channel and water flowed along the channel for washing (Figure 2(b),* rightmost*). This step should be particularly important to minimize contamination in the culture applications because the working liquids (represented by dyes here) may include abundant nutrients for growth of contaminants. In this experiment, we utilized the customized graphical user interface program to automate the entire liquid injections with a preset script loaded into the program to define the working procedures, including timing and sequences. We successfully generated a “checker” pattern in the multichamber device using the automated system as shown in Figure 2(c). In essence, this experiment has demonstrated that the accessibility of the chambers is sufficient to support the general liquid insertion operations, which require a selected liquid flowing into any of the chamber regions at different time points in defined sequences.

### 3.3. Liquid Replacement in a Microchamber

In the parallel operations, liquids such as culture media, drugs, and reagents are typically injected into the culture chambers regularly and in prescheduled sequences. In the multichamber device, liquids flow into chambers via the upstream inlet microchannels driven by a microfluidic pump [30] fabricated along the outlet channel. For higher flow rates, liquids can also be perfused into the chambers by applying a gas pressure (supplied by a compressed air source with a pressure regulator) to push the liquid to flow forward. Because of the characteristic micron-scale dimensions of microchannels and microchambers in the device, the liquid flow had a very low* Reynolds* number (Re < 1); thus the velocity profiles of flows were mostly parabolic; that is, the flow at the channel center regions was faster than the flow at some regions near the channel/chamber walls. In practice, this effect made the prediction of the amount of liquid maintained in the chamber for a certain time period of liquid injection to be difficult.

**Figure 3:**
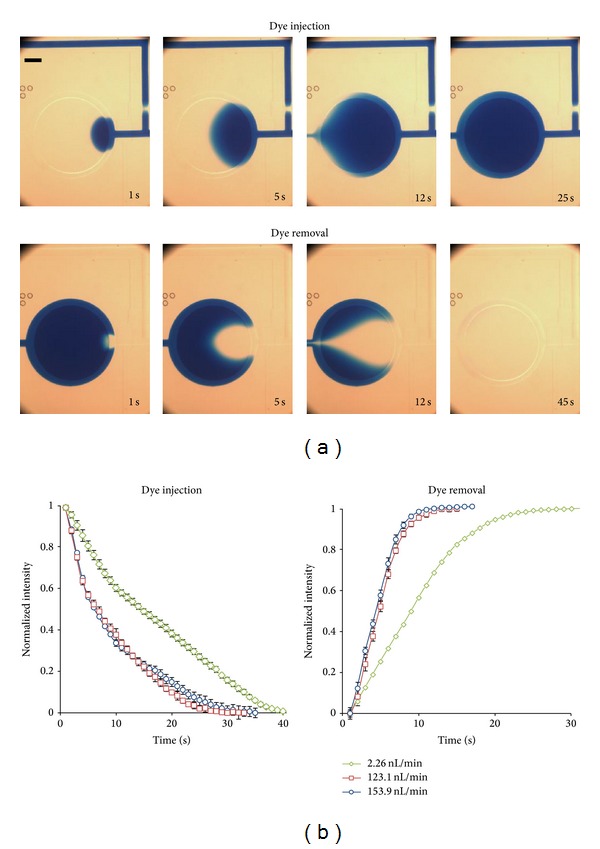
(a) Representative snapshots of dye injection (*upper* row) and dye extraction (*lower* row) processes at 1 s, 5 s, and 12 s (*left* to* right*) under a flow rate of 24.6 nL/min. Scale bar: 250 *μ*m. (b) Normalized average color intensities in the chamber for flow rates 24.6 nL/min, 123.1 nL/min, and 153.9 nL/min during the dye injection (*left*) and dye extraction (*right*) processes. All of the curves of the color intensities were obtained by at least three independent experiments. Error bars represent the standard deviations.

We performed experiments to characterize the liquid replacement process for different liquid flow rates (24.6 nL/min, 123.1 nL/min, and 153.9 nL/min). As shown in Figure 3(a), we flowed color dye and water (*transparent*) alternatively along one chamber of the device and then recorded the color change in the chamber over time. For each specified flow rate, microscopic videos for both the water-dye and dye-water processes were captured with at least three repeated experiments. We have written a customized MATLAB script to quantify the intensity changes in the chamber area and the results are summarized in Figure 3(b). It can be expected that the time for liquid replacement, indicated by the chamber color change, is shorter for flows with a higher flow rate. In essence, these experimental results provide the required time of injection for a target ratio of liquid replacement at different flow rates. Such information can help to determine the opening time of the culture chambers for different operations.

### 3.4. Image-Based Position Alignment

**Figure 4:**
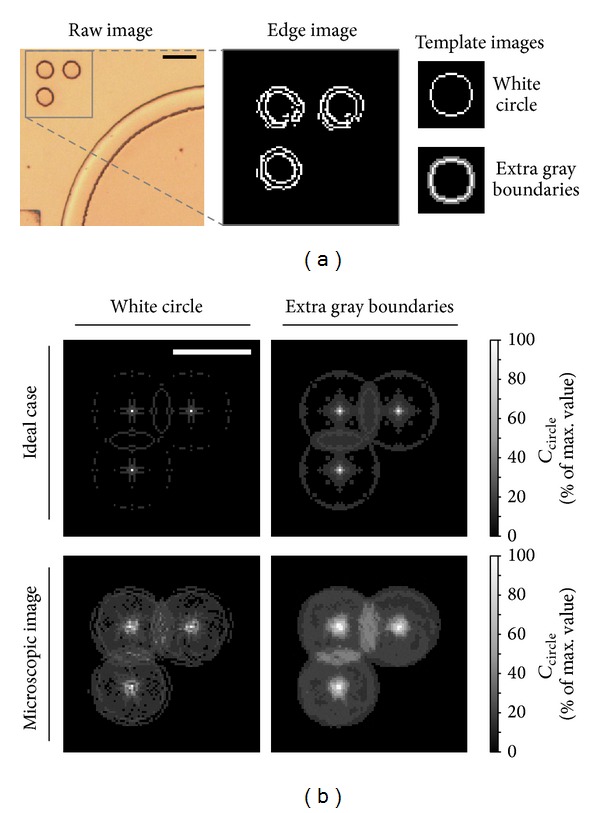
Defined geometry of the alignment mark. (a) Position of alignment mark next to a culture chamber (*left*), extracted edges of the alignment mark (*middle*), and two template images used in the convolution for alignment mark detection (*right*) in the template-matching procedures. Scale bar: 100 *μ*m. (b) Generated *C*_circle_ maps of different matching schemes between the template image and the alignment mark image. We show here a captured microscopic image (*lower*) and an ideal-case image (*upper*) of the alignment mark. Scale bar: 150 *μ*m.

The precise movements of the motorized stage alone might not guarantee the device position in the camera. Dislocation of the device on the mounting compartment of the motorized stage could be caused by loosen fixation of the mounting compartment, vibration caused by the stage, pulling force induced by the external tubing connecting to the device inlets, and so forth. Here, we designed and fabricated an alignment mark located near each chamber at a fix distance between the chamber and the mark itself. The image processing schemes were also developed in order to identify the chamber/alignment mark position and automate the continuous chamber inspection effectively. It should be mentioned that the addition of alignment marks would induce only minor additional complexity of the device fabrication for an extra layer of mold structure fabrication. An alignment mark included three circles which are formed as a right angle triangle as shown in Figure 4(a) (*left*). This pattern induced significant success rate of the position detection due to the circular pattern that was sensitive to its position rather than the orientation, which was then indicated by the arrangement of the three circles. This configuration allowed the system to detect the shifted position of every chamber via image processing, followed by automatically moving the device in position to recalibrate the viewing area.

The predefined distances between chambers allowed the position control system to switch the viewing area back and forth by moving the motorized* xy* stage chamber-by-chamber during the inspection process. After detecting the alignment mark, the displacement between target and actual positions of the alignment mark was computed, followed by movement of the* xy* stage realigning to the correct position. This realignment process could be performed repeatedly until the device was aligned (i.e., position error ≤ 3 pixels or ~3 *μ*m). In most cases, only one realignment operation was required using the microfluidic system reported in this paper. On the other hand, the moving distances between chambers were also updated during the alignment process to eliminate any errors in the predefined chamber-to-chamber distances.

#### 3.4.1. Noise Removal and Edge Extraction

We applied the Gaussian lowpass filter to first eliminate intensity noise (possibly caused by hardware of the camera sensor) in a color image, in which each pixel would have intensities in red (*R*(*x*, *y*)), green (*G*(*x*, *y*)), and blue (*B*(*x*, *y*)), where *x* and *y* represent pixel locations in the horizontal and vertical directions, respectively. Clearly, the pixel brightness *I*_*O*_(*x*, *y*) can be calculated by *I*_*O*_(*x*, *y*) = [*R*(*x*, *y*) + *G*(*x*, *y*) + *B*(*x*, *y*)]/3. We then performed the Gaussian lowpass filter by convolution of the captured image with a Gaussian mask *G*(*i*, *j*), which is a 2D gray-scale image (size: *D* × *D*) expressed as

(1) $$\begin{array}{c} G\left(i,j\right)=\frac{1}{2N\pi \sigma^{2}}\cdot e^{-(\left(i^{2}+j^{2}\right)/2\sigma^{2})}, \end{array}$$

where *σ* is standard deviation of the Gaussian function; *N* = *D* ^2^ is the number of pixels in the Gaussian mask; *i* and *j* are pixel locations in the horizontal and vertical directions in the mask, respectively. It should be noted the pixel located at *i* = 0 and *j* = 0 represents the center of the mask. Hence, the pixel gray-scale intensity *I*_*G*_(*x*, *y*) after the convolution is

(2) IG(x,y)=∑i=−(D−1)/2(D−1)/2 ∑j=−(D−1)/2(D−1)/2G(i,j)IO(x+i,y+j),∀x  and  y  in  the  image.

*x* and *y* are the pixel positions in the image in the horizontal and vertical directions, respectively. Thus, *x* and *y* are both integers between “1” and *D*.

We then applied the* Sobel* edge operator to recognize the edges in an image. Edge detection was an essential step to extract key features of the alignment mark. Similar to the Gaussian filter, convolution was utilized to calculate the intensity gradients at the processing pixel *S*_*x*_(*x*, *y*) and *S*_*y*_(*x*, *y*) in *x* and *y* directions, respectively:

(3) $$\begin{array}{c} S_{x}\left(x,y\right)=\overset{1}{\underset{i=-1}{\sum }}\;\overset{1}{\underset{j=-1}{\sum }}I_{G}\left(x+i,y+j\right)M_{x}\left(i,j\right),\\ S_{y}\left(x,y\right)=\overset{1}{\underset{i=-1}{\sum }}\;\overset{1}{\underset{j=-1}{\sum }}I_{G}\left(x+i,y+j\right)M_{y}\left(i,j\right), \end{array}$$

where *M*_*x*_ and *M*_*y*_ are masks of* Sobel* edge operators for the horizontal and vertical directions, respectively:

(4) $$\begin{array}{c} M_{x}=\left(\begin{array}{ccc} -1 & 0 & 1\\ -2 & 0 & 2\\ -1 & 0 & 1 \end{array}\right),\;\;M_{y}=\left(\begin{array}{ccc} 1 & 2 & 1\\ 0 & 0 & 0\\ -1 & -2 & -1 \end{array}\right). \end{array}$$

We considered the magnitude of the total gradient* S*(*x*,* y*), approximated by

(5) $$\begin{array}{c} S\left(x,y\right)=\sqrt{S_{x}{\left(x,y\right)}^{2}+S_{y}{\left(x,y\right)}^{2}}\approx \left|S_{x}\left(x,y\right)\right|+\left|S_{y}\left(x,y\right)\right|. \end{array}$$

We further processed *S*_*x*_(*x*, *y*) and *S*_*y*_(*x*, *y*) at each pixel to determine the angle of the intensity gradient *θ*(*x*, *y*) by computing *θ*(*x*, *y*) = tan^−1^⁡[*S*_*y*_(*x*, *y*)/*S*_*x*_(*x*, *y*)]. We considered that a pixel was an edge point when its corresponding *S*(*x*, *y*) was larger than the magnitudes of the total gradients of the neighboring pixels along the direction of *θ*(*x*, *y*). To simplify the computation, *θ*(*x*, *y*) was rounded-off to the nearest value as a multiple of 45° (0°, ±45°, ±90°, ±135° and 180°). In this step, a “prethinned” edge image *S*_*E*_′(*x*, *y*) was generated with an edge pixel in* white* and a nonedge pixel in* black* (zero intensity). The image thinning process was applied afterward such that the edge image *S*_*E*_(*x*, *y*) included its most edges around one pixel wide (Figure 4(a),* middle*).

#### 3.4.2. Detection of Alignment Mark Position

Alignment marks in a captured image were then detected by a template-matching approach. Briefly, positions of the circular patterns were detected by the pixel with a maximum value after the convolution between the processed edge image and the template image, *I*_*T*_(*x*, *y*) (size: *D*_*T*_ × *D*_*T*_). We defined two circular pattern templates which included pixels with* white*,* gray,* or* black* colors as shown in Figure 4(a) (*right*). The* black* pixels represented the excluded regions and they were ignored during the image convolution step. The* white* and* gray* pixels indicated the image template pattern. More specifically, one temple had only a* white* circle to match the outer edges of the processed circle patterns microscopic image; while the other template included also the outer and inner* gray* boundaries to improve the detection accuracy by concerning the fact that positions of the edge pixels from the edge detection operations may have round-off errors in the practical implementations. The convolution of these gray pixels still covered the possible displaced edge pixels of the circular patterns. We now express the image generated after convolution of the edge image and the template image *C*_circle_ as

(6) Ccircle(x,y)=∑j=0DT ∑i=0DTSE(x+i,x+j)IT(i,j)∑j=0DT ∑i=0DTIT(i,j),∀IT(i,j)  in  the  pattern.

*C*_circle_(*x*, *y*) reflects the degree of matching between the edge image and the template image at the pixel position (*x*, *y*). Therefore, the three maximum points of *C*_circle_(*x*, *y*) should indicate the position of circle patterns.

We conducted experiments to demonstrate the distributions of *C*_circle_(*x*, *y*) using the two template images: (1) only a white circle and (2) a white circle with one-pixel thick gray boundaries around the circle. We first computed the *C*_circle_ maps for the template-matching on an ideal alignment mark, which contained three circle patterns identical to the white-circle template pattern (*upper* maps in Figure 4(b)). Both template patterns could precisely identify the circle pattern locations. The gray-boundary template induced transition regions around the maximum *C*_circle_ values. We further tested performance of the template-matching scheme on the microscopic image shown in Figure 4(a). It can be observed that using the white-circle template could induce multiple bright pixels around the circle centers in the *C*_circle_ map. This characteristic made a precise position detection of the circle patterns to become challenging. For the case using the gray-boundary circle, the outcome *C*_circle_ map showed a more reasonable profile of the values where the brightness regions were all at the circle centers. Hence, the gray-boundary template image should achieve a more robust position identification of the circle patterns using the template-matching approach.

Subsequently, positions of the three circle patterns were detected by finding the three maximum values in *C*_circle_(*x*, *y*) using the gray-boundary template image for matching. We defined the positions of these circles as (*x*_*c*1_, *y*_*c*1_), (*x*_*c*2_, *y*_*c*2_), and (*x*_*c*3_, *y*_*c*3_) for the* upper left*,* right*, and* lower* circles, respectively. Hence, we could set the reference position of the alignment mark as the position of the* upper left* circle, that is, (*x*_*c*1_, *y*_*c*1_). We estimated the mark orientation as {tan^−1^⁡[(*y*_*c*2_ − *y*_*c*1_)/(*x*_*c*2_ − *x*_*c*1_)] + tan^−1^⁡[(*y*_*c*1_ − *y*_*c*3_)/(*x*_*c*1_ − *x*_*c*3_)] − 90°}/2. Thereby, the center of a chamber can be aligned with a target position based on these measured displacements and orientations of the alignment mark accordingly.

#### 3.4.3. Example of the Position Alignment Procedures

**Figure 5:**
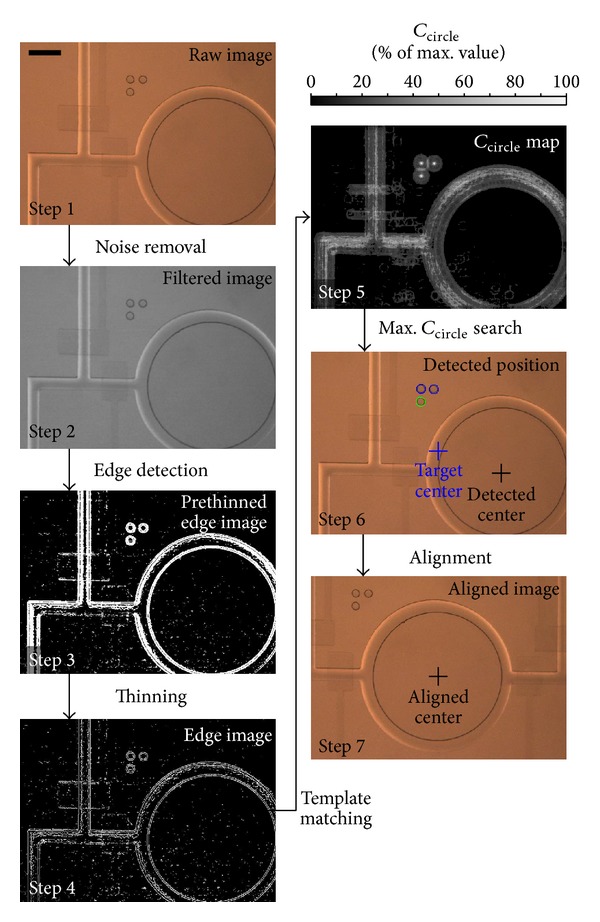
Different steps during the automated chamber alignment procedures. Scale bar: 250 *μ*m.

Figure 5 illustrates typical outcomes of the steps during the image-based alignment process. Most of the intensity noise in a representative raw microscopic image was removed by the Gaussian lowpass filtering. Edges of the filtered image were extracted by the* Sobel* edge operators, followed by the thinning operation for the estimation of finer edges. Afterwards, the map of *C*_circle_(*x*, *y*) was generated by the template-matching process and positions of the three circles in the alignment marks were indicated with the maximum values in *C*_circle_(*x*, *y*). Based on these circle position, the required movement of the *xy* stage was computed and therefore the chamber was aligned with the target location for time-lapse recording and further analyses.

### 3.5. Characterization of Automated Position Alignment

**Figure 6:**
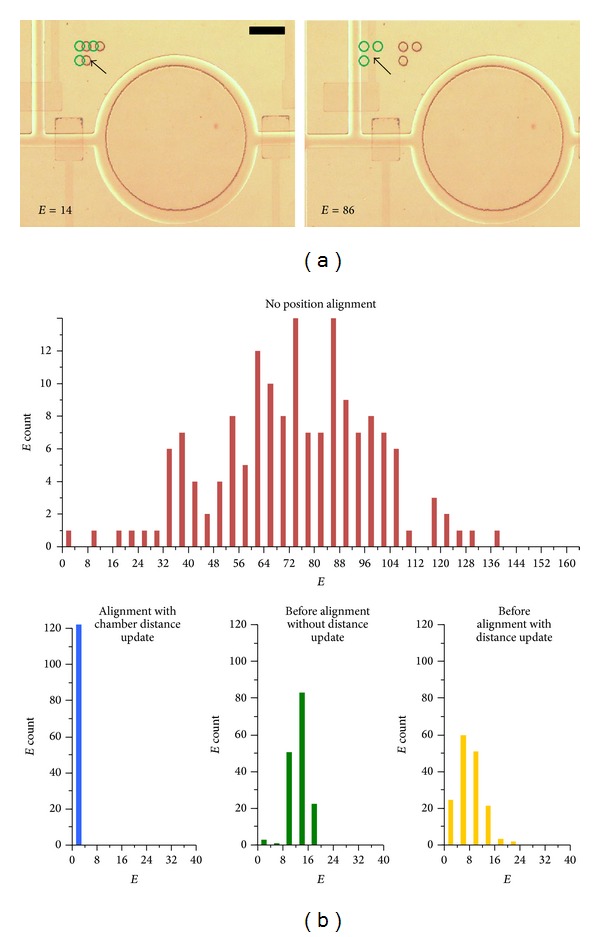
(a) Representative micrographs for two different position errors:* E* = 14 (*left*) and* E* = 86 (*right*). Arrows indicate the target positions of alignment marks. Scale bar: 250 *μ*m. (b) Statistics of position errors for different cases throughout the automation chamber alignment procedures.

Multiple steps of chamber-to-chamber movements of the *xy* stage were performed to examine the importance for different stages during the position alignment process. The position error of a chamber (*E*, unit: pixel) was defined as the distance between the target and detected alignment mark positions. For instance, Figure 6(a) shows that the value of *E* can describe effectively the level of misalignment of the device. We performed experiments to obtain the *E* values for four conditions: (1) without automated position alignment, (2) after alignment with chamber distance update (step 7 in Figure 5), (3) before alignment without chamber distance update, and (4) before alignment with chamber distance update (step 1 in Figure 5). Each of these cases included >80 independent measurements. As shown in Figure 6(b), considering that the pixel width is 0.94 *μ*m, the distributions of *E* were 73.6 ± SD24.8 pixels (or 69.2 ± SD23.3 *μ*m;* upper*), 1.03 ± SD0.65 pixels (or 0.97 ± SD0.61 *μ*m;* lower left*), 7.97 ± SD4.00 pixels (or 7.5 ± SD3.7 *μ*m;* lower middle*), and 14.1 ± SD10.1 pixels (or 13.3 ± SD9.5 *μ*m;* lower right*) for the above cases 1–4, respectively. These results show that the automated alignment operation greatly reduces the position errors from ~74 pixels (~69.2 *μ*m) to ~1 pixel (0.94 *μ*m). During the alignment process, each time of the stage movement to a nearby chamber can induce an average position error of ~7 pixels (~6.6 *μ*m). Comparing the *E* values before alignment operations with (case 3) and without (case 4) the chamber distance update, we can observe that the distance update reduces *E* due to the preset stage moving distances. This implies that fewer iterative operations should be required for the position alignment and the overall processing time can then be shortened.

**Figure 7:**
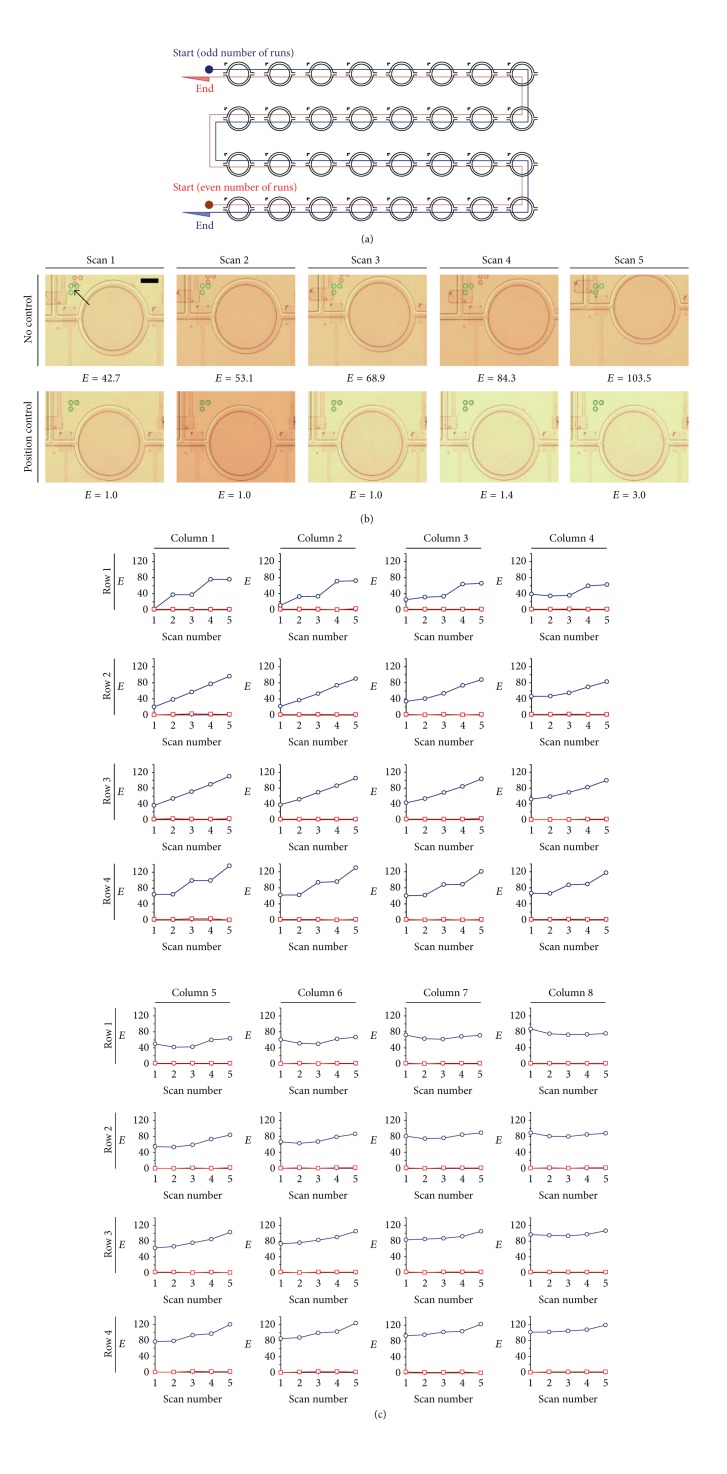
Positioning errors of chambers (*E*) during five times of scans. (a) Sequence of odd-number scan and even-number scan. (b) Image representation of errors for the chamber at row 3 and column 3. The arrow indicates the target location of alignment marks. With the position control, all alignment marks were maintained at the target locations. Scale bar: 250 *μ*m. (c)* E* values of the 32 chambers during the 5 scans. Circles and squares represent the conditions without control and with position control, respectively.

On the other hand, we investigated also variations of the shifted positions of all of the chambers after* xy* stage moving repeatedly with and without the position alignment. The system recorded the *E* values during multiple scans of imaging over the 32 chambers in the device. Paths of the scans were implemented by the motorized* xy* stage moving back and forth as described in Figure 7(a). We wrote scripts for the graphical user interface program to define sequences of the movements of the stage and the imaging operations as well as the moving distances between chambers. Here, we show in Figure 7(b) the sample images of a microchamber (at row 3 and column 3 in the device) for its position errors (*E*) during five imaging scans. Apparently, the *E* was maintained by the position control operations, while the *E* without the control accumulated with the numbers of scans. Indeed, the similar trend happened also in all other chambers as shown in Figure 7(c). After the five scans,* E* values for the uncontrolled case were ~2–80 times larger than the *E* values with the realignment. Moreover, the *E* values of the outer chambers were in general larger. This observation implies that further integration of the microfluidics with more chambers (i.e., a larger chip size) should have an even larger demand for the automated alignment function.

### 3.6. Implementation of Mammalian Cell Culture

**Figure 8:**
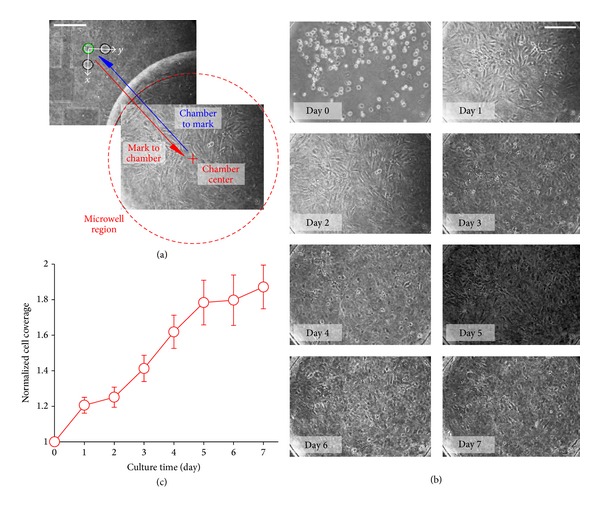
Implementation of automated monitoring of parallel cell culture. (a) Switch between cell alignment mark and chamber center for imaging. Scale bar: 250 *μ*m. (b) Example of cell seeding (day 0) and cell growth (day 1–7) in a representative chamber. Scale bar: 250 *μ*m. (c) Normalized cell coverage in microchambers of the microfluidic device. Error bars represent the standard errors with each calculated from 5–20 data points.

To demonstrate potentials of the automated parallel monitoring strategy in cell research applications, we cultivated BALB murine embryonic fibroblast cells (3T3) in all of the 32 chambers in the microfluidic device over one week until a confluent cell density was observed in all of the chambers. Cells were first seeded into each chamber and maintained in the growth condition (37°C and 5% CO_2_ in humidified air) for 8 hr to facilitate the cell attachment and spreading. During the cell culture, media in chambers were replaced for every 4 hr. Images were then taken at all of the chambers regularly. As described in Figure 8(a), we adopted a position-shifting scheme between the alignment mark and the chamber center in order to maximize the regions of the chamber for the automated imaging. In more details, the motorized stage would first align with the corresponding target alignment mark (*green* circle) with the aid of the presented automated alignment scheme. The observation position would then shift from the alignment mark to the chamber center for 1.2 mm in both vertical (*x*−) and horizontal (*y*−) directions of the microfluidic device (*red* arrow), followed by the imaging step. It should be mentioned that this single step of stage movement with a short distance did not induce any significant misalignment during the implementation. The observation position then shifted back to the alignment mark (*blue* arrow) for the alignment with the next selected chamber. As a representative result of the cell growth in the microfluidic device, microscopic images of the cell population in a chamber at different time points during the experiment are shown in Figure 8(b). Images for cell growths in all of the 32 chambers of the microfluidic devices are also available in Figure S4. Results indicated that cell growth could be maintained in the microfluidic device for at least 7 days. We quantified the cell densities at different time points (each with 5–20 repeated experimental values) using image processing software (ImageJ, National Institutes of Health) as shown in Figure 8(c). Essentially, these results indicate that the reported microfluidic platform can regulate microenvironments for cell proliferation and can support further parallel cell analyses and monitoring applications using high-throughput microfluidics.

## 4. Conclusion

In this work, we developed a microfluidic position control system capable of implementing microfluidic manipulation and automated image-based microchamber alignment. The system included also temperature and gas control to regulate the desired microenvironment for cell or chemical applications. We designed and fabricated an integrated microfluidic device containing 32 chambers with the alignment marks, which were composed of three circular patterns. The addition of these alignment marks did not induce any extra fabrication efforts. The chamber accessibility and fluidic manipulation were implemented by the configuration of channels and control valves in the device. We characterized the required durations for liquid replacement in the chambers under different flow rates. We demonstrated also that each chamber in the device can be flowed with a liquid selected from the inlets. On the other hand, we applied image processing techniques (e.g.,* Sobel* edge detector and temple matching scheme) on the alignment mark regions to identify the chamber positions and their dislocations from the target locations. We implemented hardware acceleration to shorten the overall processing time of the alignment mark detection from 20389 ms to 452 ms (~2.2%). The effectiveness of the automated chamber alignment was shown by our experiments where position errors for the unaligned case were ~2–80 times larger than errors for the aligned case in every scan of the 32 chambers. The errors for the unaligned case accumulated throughout multiple scans, whereas the errors for the aligned case were maintained within 3 pixels. Further, we demonstrated also that the microfluidic parallel monitoring platform can be applied to cell growth analyses. Altogether, in view of the fact that the alignment marks can be embedded in any microfluidic designs, this automated image-based chamber alignment can be applied in the general high-throughput microfluidics for continuous monitoring of cell and biochemical activities at multiple regions simultaneously.

## Supplementary Material

This Supplementary Material includes additional figures describing more detailed information of the microfluidic automation platform, such as consistency of the temperature profile and gas flow around an operating microfluidic device. It also provides a graphical presentation of the device fabrication and representative raw images of the cells growing in multiple wells of the device.
