# Supplementary material for: Automated Long-Term Monitoring of Parallel Microfluidic Operations Applying a Machine Vision-Assisted Positioning Method
Source: ScientificWorldJournal. 2014 Jul 15;2014:608184. doi: 10.1155/2014/608184 (PMC4124227; doi:10.1155/2014/608184)
Supplement: Supplementary file 1 — This Supplementary Material includes additional figures describing more detailed information of the microfluidic automation platform, such as consistency of the temperature profile and gas flow around an operating microfluidic device. It also provides a graphical presentation of the device fabrication and representative raw images of the cells growing in multiple wells of the device. [file 608184.f1.pdf]

Automated Long-Term Monitoring of Parallel Microfluidic Operations Applying a Machine Vision-Assisted Positioning Method

Hon Ming Yip, John C. S. Li, Kai Xie, Xin Cui, Agrim Prasad, Qiannan Gao, Chi Chiu Leung and Raymond H. W. Lam

Department of Mechanical and Biomedical Engineering
City University of Hong Kong, Hong Kong

* Correspondence should be addressed to R. H. W. Lam; email: rhwlam@cityu.edu.hk; Tel: 852-3442-8577; Fax: 852-3442-0172.

SUPPLEMENTAL FIGURES

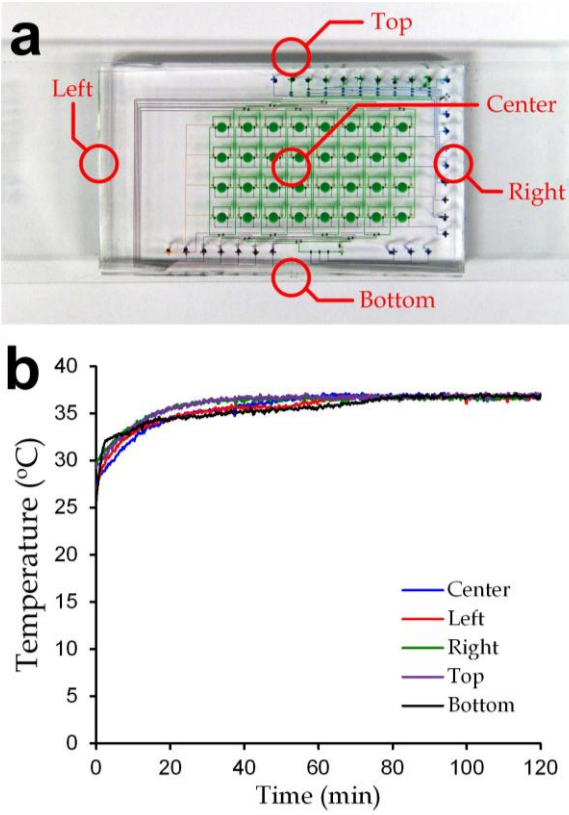

Figure S1. (**a**) Positions of temperature sensor placement on the microfluidic device mounted on the microscope. The temperature measurements were then performed to analyze for homogeneity of the device temperature during operation. For the measurements, we used a temperature sensor (LM35, Texas Instrument) connected with our data acquisition board controlled by a microprocessor (Atmega16, Atmel Corporation). The board then passes the temperature values to a computer via the serial communication. (**b**) Temperature profiles at the selected position of measurement. The device temperature should become homogenous at the target level within 90 min of the temperature. Therefore, it is suggested that the temperature control should be run for 90 min before experiments for a stabilized level.

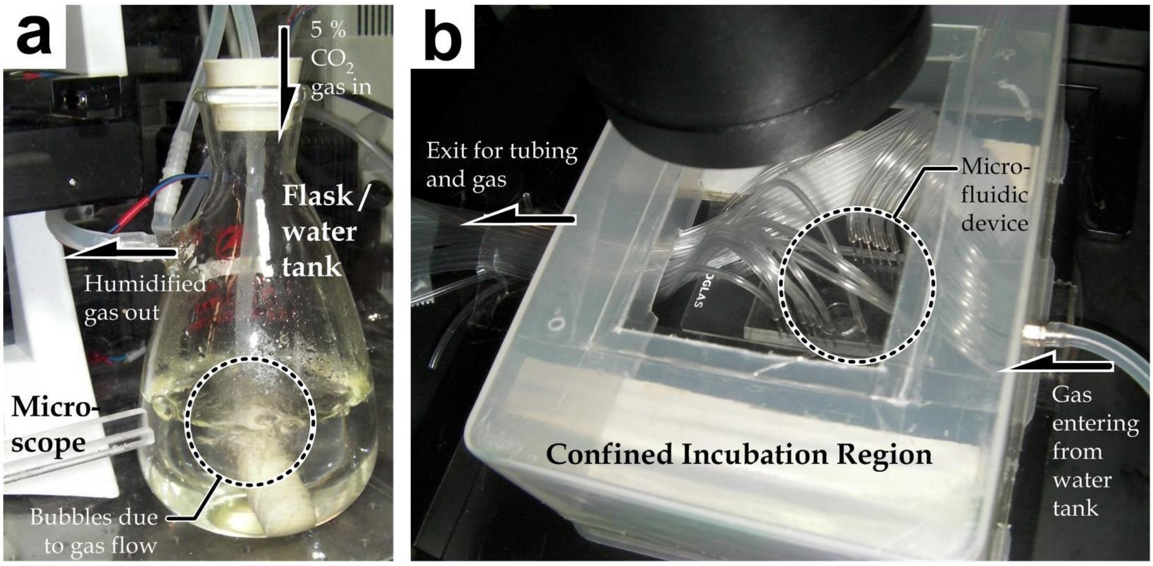

Figure S2. Photographs of water tank and confined incubation region. (**a**) The water tank included a flask filled with distilled water for humidification of compressed air with 5 % CO_2. The gas flowed through the water and then to the confined incubation region through tubing. The lengths of tubing placed in the temperature controlled shield were >1 m for both upstream and downstream of the water tank, because a sufficient traveling time of the flowing gas should be provided for temperature stabilization. (**b**) The confined incubation region contained a microfluidic cell culture device was place in the region. It included also a gas inlet on one side, and an opening on the opposite side for the tubing insertion and the gas exit.

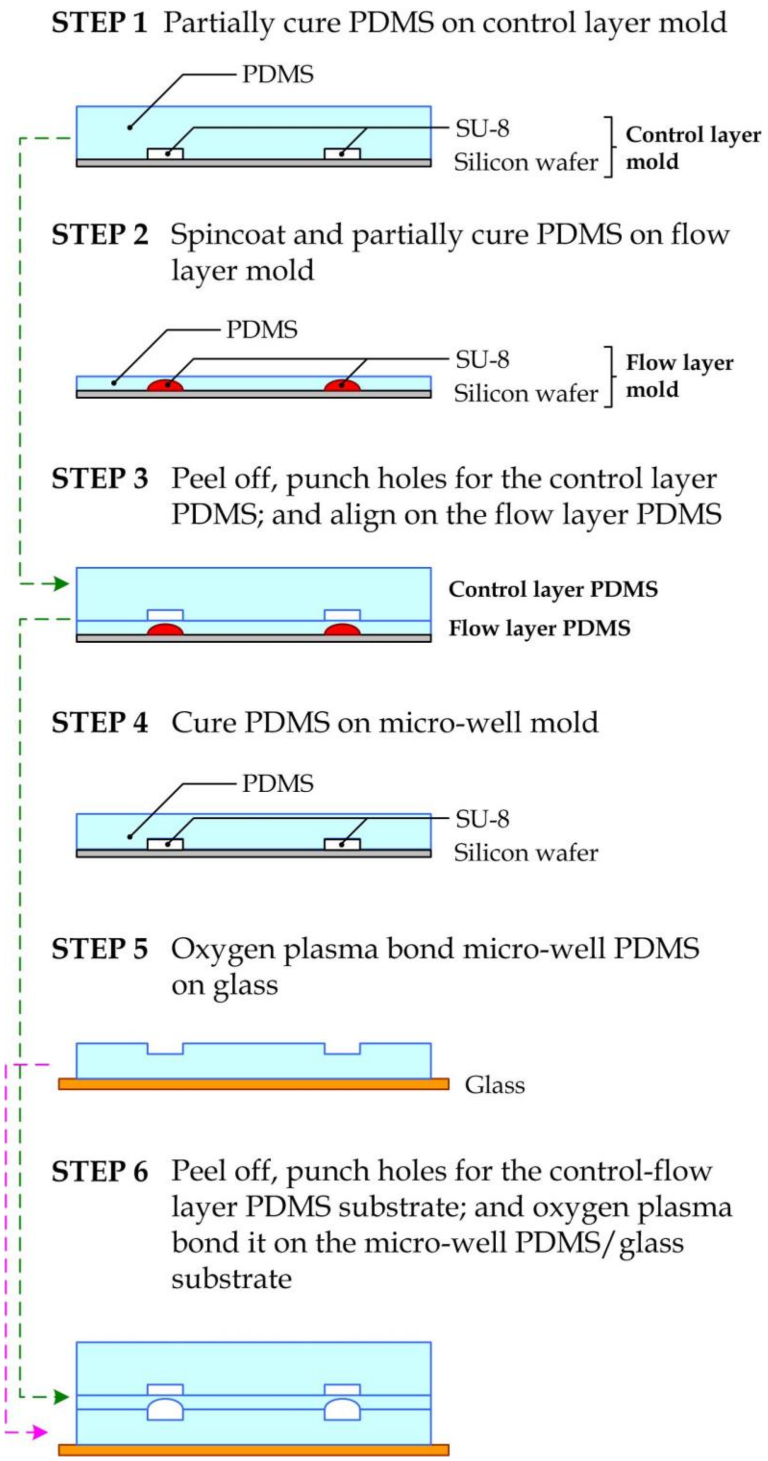

Figure S3. Illustration of the microfluidic device fabrication based on soft lithography.

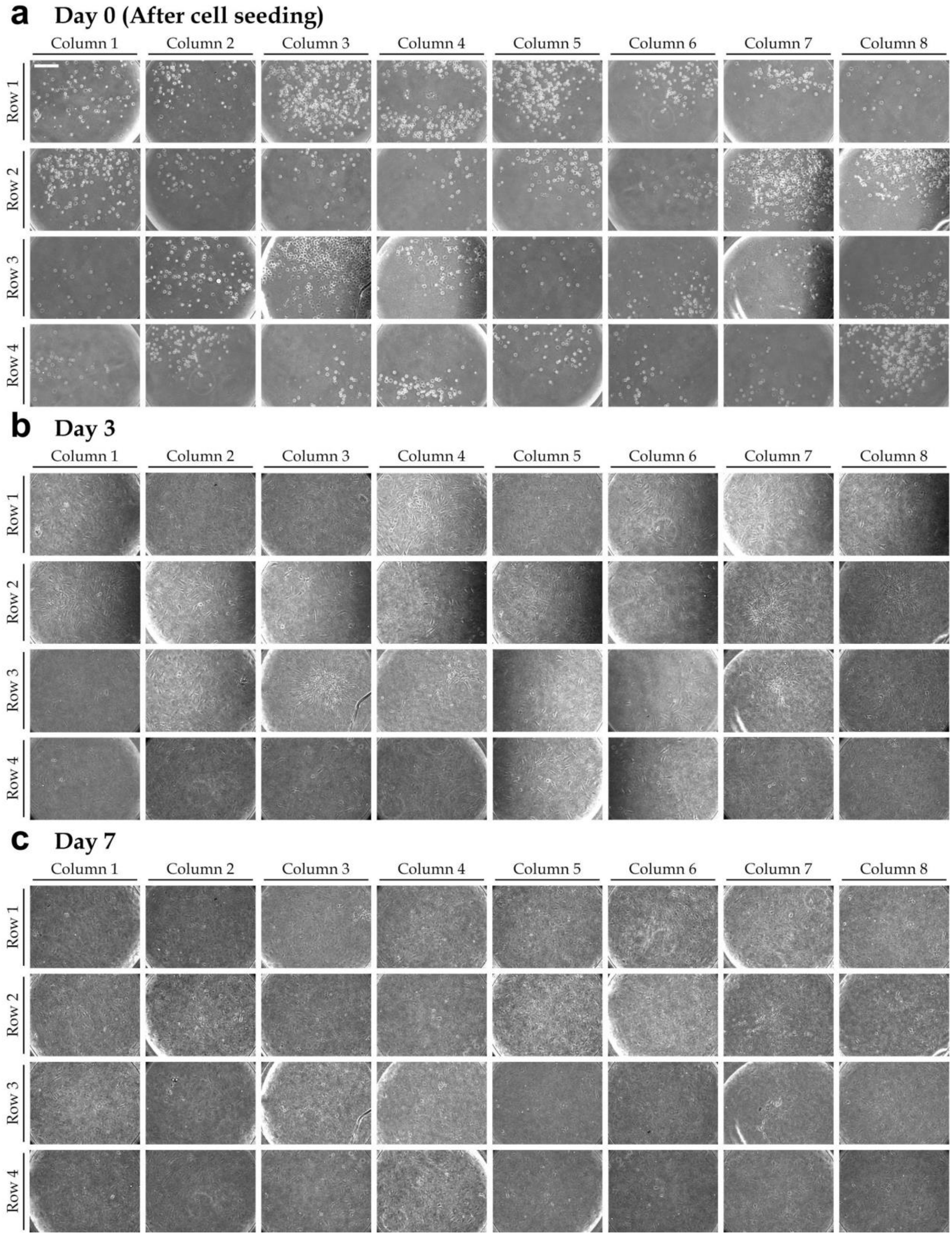

Figure S4. Images of cell populations in the 32 chambers of the microfluidic device after (**a**) cell seeding, (**b**) 3 days of culture and (**c**) 7 days of culture. These images were all taken at different time points using the image-assisted positioning algorithm for the automated imaging process. Scale bar in the *upper-left* micrograph of (**a**): 400 μm.
